# Characterization of single-stranded DNA-binding proteins from the psychrophilic bacteria *Desulfotalea psychrophila*, *Flavobacterium psychrophilum*, *Psychrobacter arcticus*, *Psychrobacter cryohalolentis*, *Psychromonas ingrahamii*, *Psychroflexus torquis,* and *Photobacterium profundum*

**DOI:** 10.1186/1471-2180-14-91

**Published:** 2014-04-14

**Authors:** Marta Nowak, Marcin Olszewski, Marta Śpibida, Józef Kur

**Affiliations:** 1 Department of Microbiology, Faculty of Chemistry, Gdańsk University of Technology, ul. Narutowicza 11/12, 80-233 Gdańsk, Poland

**Keywords:** DNA replication, Expression, Psychrophilic microorganism, SSB, Thermostability, Psychrophiles

## Abstract

**Background:**

Single-stranded DNA-binding proteins (SSBs) play essential roles in DNA replication, recombination and repair in Bacteria, Archaea and Eukarya. In recent years, there has been an increasing interest in SSBs, since they find numerous applications in diverse molecular biology and analytical methods.

**Results:**

We report the characterization of single-stranded DNA-binding proteins from the psychrophilic bacteria *Desulfotalea psychrophila* (*Dps*SSB), *Flavobacterium psychrophilum* (*Fps*SSB), *Psychrobacter arcticus* (*Par*SSB), *Psychrobacter cryohalolentis* (*Pcr*SSB), *Psychromonas ingrahamii* (*Pin*SSB), *Photobacterium profundum* (*Ppr*SSB), and *Psychroflexus torquis* (*Pto*SSB). The proteins show a high differential within the molecular mass of their monomers and the length of their amino acid sequences. The high level of identity and similarity in respect to the *Eco*SSB is related to the OB-fold and some of the last amino acid residues. They are functional as homotetramers, with each monomer encoding one single stranded DNA binding domain (OB-fold). The fluorescence titrations indicated that the length of the ssDNA-binding site size is approximately 30 ± 2 nucleotides for the *Pin*SSB, 31 ± 2 nucleotides for the *Dps*SSB, and 32 ± 2 nucleotides for the *Par*SSB, *Pcr*SSB, *Ppr*SSB and *Pto*SSB. They also demonstrated that it is salt independent. However, when the ionic strength was changed from low salt to high, binding-mode transition was observed for the *Fps*SSB, at 31 ± 2 nucleotides and 45 ± 2 nucleotides, respectively. As expected, the SSB proteins under study cause duplex DNA destabilization. The greatest decrease in duplex DNA melting temperature was observed in the presence of the *Pto*SSB 17°C. The SSBs in question possess relatively high thermostability for proteins derived from cold-adapted bacteria.

**Conclusion:**

The results showed that SSB proteins from psychrophilic microorganisms are typical bacterial SSBs and possess relatively high thermostability, offering an attractive alternative to other thermostable SSBs in molecular biology applications.

## Background

Single-stranded DNA-binding proteins (SSBs) are indispensable elements in the cells of all living organisms. They interact with ssDNA regardless of sequence, preventing them from forming secondary structures and protecting them from degradation by nucleases [1]. In this way, they participate in all the processes involving ssDNA, such as replication, repair and recombination [2-5]. Although there are differences in amino acid sequences, SSBs have a high-conservative domain, the oligonucleotide/oligosaccharide–binding fold, referred to as the OB-fold, which is responsible for binding with ssDNA [6]. In the single-stranded DNA-binding proteins described so far, four OB-fold domains form an active protein. These proteins also have the ability to bind RNA and are present in all three branches of live organisms and in viruses. The cooperative binding of single-strand DNA and RNA, which is a property of SSBs, has led to their being used as tools in molecular biology methods and analytics. Thermostable proteins are particularly useful in this respect. To date, only a few thermostable SSB proteins with these valuable applications have been identified.

Information resources on proteins from cold-adapted microorganisms are extremely limited, particularly when the spread of psychrophilic organisms in the environment is taken into account; approximately 85% of the Earth’s Biosphere is an environment with temperatures of below 5°C.

We recently presented a study on the production, purification and molecular characteristics of a single-stranded DNA binding protein from *Pseudoalteromonas haloplanktis*, the first report of a protein of this kind from a psychrophilic microorganism [7]. The proteins which are the focus of interest in this article come from different phylogenetically-related obligate and facultative psychrophilic Gram-negative bacteria. *Photobacterium profundum* str. SS9, which belongs to Gammaproteobacteria, Vibrionaceae family, was isolated from the Sulu Trough associated with Amphipoda at a depth of 2551 m. It is a psychrophilic and moderately barophilic bacterium with an optimum growth temperature and pressure of 15°C and 20 MPa, respectively [8]. *P. profundum* SS9 is a genetically tractable model system for studies of low-temperature and high-pressure adaptation [9]. *Desulfotalea psychrophila*, which belongs to Deltaproteobacteria, Desulfobulbaceae family, is a sulfate-reducing bacteria isolated from permanently cold Arctic sediments off the coast of Svalbard, Norway [10]. *Flavobacterium psychrophilum*, belongs to Bacteroidetes, Flavobacteriaceae family, is a facultative psychrophilic bacterium and one of the most serious of the fish pathogens [11]. The *Psychrobacter arcticus* and *Psychrobacter cryohalolentis* strains, which belong to Gammaproteobacteria, Moraxellaceae family, were isolated from permafrost samples taken from the Kolyma lowland region of Siberia, Russia [12]. *P. arcticus* was a model organism for studies on the mechanisms of adaptation to low temperatures [13]. *Psychromonas ingrahamii* bacterium, which belongs to Gammaproteobacteria, Psychromonadaceae family, was isolated from a sea ice core collected on Point Barrow in Alaska, USA. The bacterium grows well at NaCl concentrations of 1-10% and at temperatures of −12 to 10°C; no growth is observed at 15°C, and the optimal growth temperature is 5°C. *Psychromonas ingrahamii* is the only bacterium growing at such a low temperature to have been described to date [14]. *Psychroflexus torquis*, which belongs to Bacteroidetes, Flavobacteriaceae family, is isolated from Antarctic sea ice psychrophilic bacterium. The representatives of this species possess an uncommon characteristic, the ability to synthesize polyunsaturated fatty acids [15].

The aim of this study was to clone and overexpress *D. psychrophila*, *F. psychrophilum*, *P. arcticus*, *P. cryohalolentis*, *P. ingrahamii*, *P. profundum*, and *P. torquis ssb*-like genes in *E. coli*, purify the gene products and study their biochemical properties.

## Results

### Sequence analysis

The sequence analysis of the *D. psychrophila* (GenBank accession No. NC_006138; [16]), *F. psychrophilum* (GenBank accession No. NC_009613; [17]), *P. arcticus* (GenBank accession No. NC_007204; [18]), *P. cryohalolentis* (GenBank accession No. NC_007969; Gene Bank Project: PRJNA58373), *P. ingrahamii* (GenBank accession No. NC_008709; [19]), *P. profundum* (GenBank accession No. NC_006370; [20]) and *P. torquis* (GenBank accession No. NC_018721; [15]) genomes indicated the presence of a single *ssb* gene. In the case of *F. psychrophilum*, *P. ingrahamii* and *P. torquis*, there were additional genes possessing sequences similar to the ssDNA binding domain. The product of the additional gene from *F. psychrophilum* was a protein of unknown function, while that from *P. ingrahamii* was the PriB. In *P. torquis*, it was a short (102 aa), single-stranded DNA binding protein without a characteristic sequence of last amino acid residues, in view of which, we omitted that protein from our research. On the basis of the *ssb* gene organization and the number of *ssb* genes paralogs, bacteria have been classified in four different groups [21]. *P. arcticus*, *P. cryohalolentis* and *P. profundum* are classified as group III, which contains bacteria with *ssb* gene organization *uvrA-ssb*, whereas *D. psychrophila*, *F. psychrophilum*, *P. ingrahamii*, and *P. torquis* are classified as group IV, which contains bacteria with *ssb* placed neither between *rpsF* and *rpsR* nor divergently located to *uvrA*.

The *Dps*SSB, *Fps*SSB, *Par*SSB, *Pcr*SSB, *Pin*SSB, *Ppr*SSB, and *Pto*SSB proteins contain 142, 140, 213, 219, 222, 183, and 151 amino acid residues, respectively, including the N-terminal methionine, as is apparent from the nucleotide sequence. Analysis of the primary structures by RPS-BLAST revealed the presence of two distinctive regions in the proteins in question: one putative OB-fold domain, from amino acid 1 to 105–110, and one C-terminal domain, which contains four conserved terminal amino acid residues common in all known bacterial SSB proteins. The molecular mass of its monomers show a high differential, ranging from 15.6 to 25.1 kDa. Besides the OB-fold, the C-terminal fragment has the characteristic of a highly differential length, ranging from 31 to 112 amino acid residues. At their ends, the C-terminal domains have amino acids which are either similar or identical to the *Eco*SSB. The computable isoelectric point in these proteins has values in the range of 5–6, which is typical for SSBs with the exception of *Pin*SSB, pI 7.79 (Table 1).

**Table 1:** Characteristics resulting from the amino acid sequence analysis of the SSB proteins under study

**SSB**	**Size of monomer [kDa]**	**Length of sequence [aa]**	**Length of C-terminal domain [aa]**	**Sequence of last important amino acid residues**	**pI**	**Aliphatic index**	**No. of Cys residues**
*Dps*SSB	15.6	142	37	DVPF	5.46	61.20	1
*Fps*SSB	15.9	140	31	DLPF	5.94	73.07	2
*Par*SSB	22.8	213	105	DIPF	5.91	49.11	0
*Pcr*SSB	23.3	219	111	DIPF	5.70	43.29	0
*Pin*SSB	25.1	222	112	DIPF	7.79	41.80	1
*Pto*SSB	17.1	151	43	DLPF	5.67	61.32	3
*PprSSB*	20.4	183	76	DIPF	5.43	54.37	0
*Eco*SSB	18.9	178	73	DIPF	5.44	56.97	0

**Figure 1:**
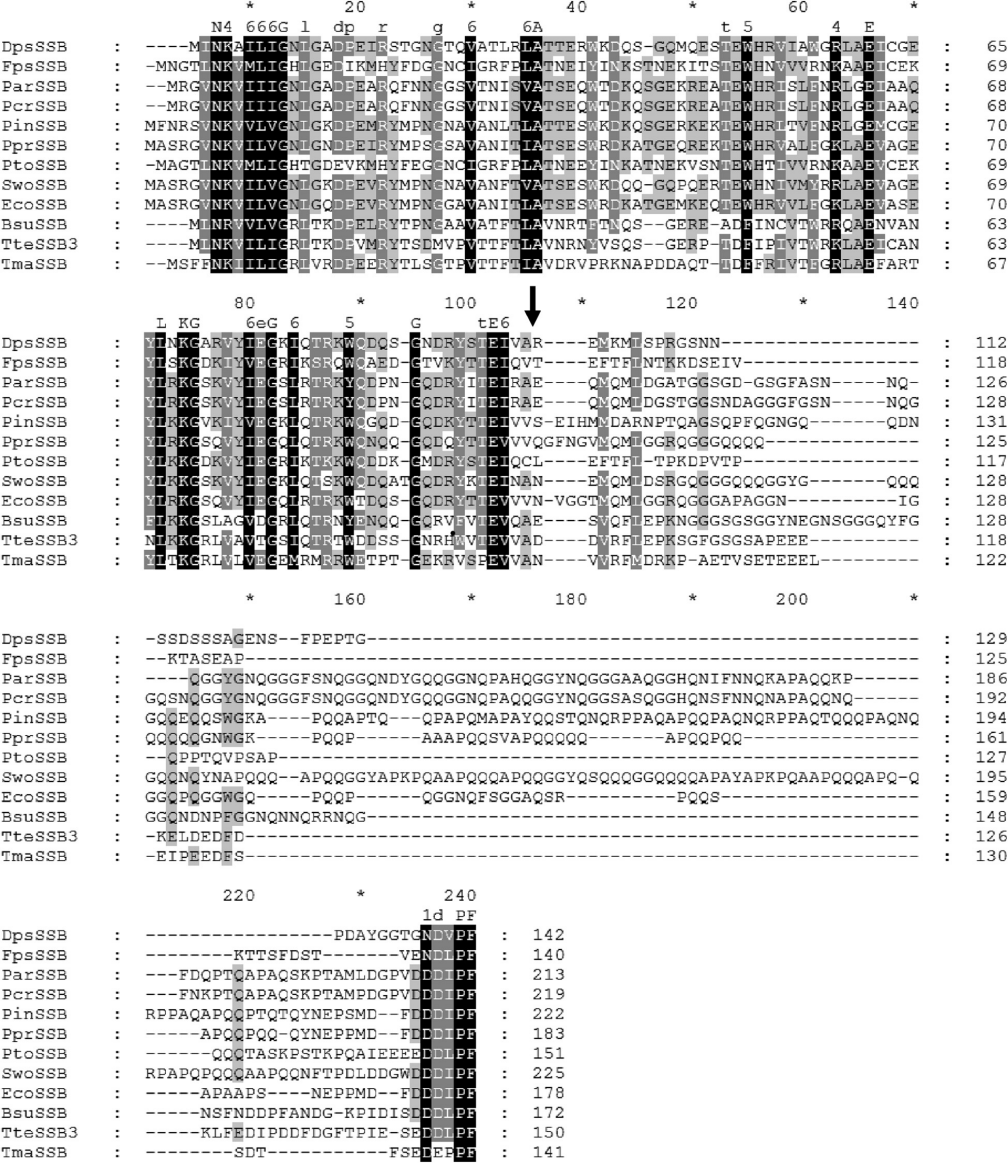
**The multiple amino acid alignment of the SSB proteins under study, with the SSBs from psychrophilic, mesophilic and thermophilic bacteria.** The alignments were performed by dividing the amino acids into six similarity groups: group 1 V, L, I, M, group 2 W, F, Y, group 3 E, D, group 4 K, R, group 5 Q, D, and group 6 S, T. The capital letters represent single amino acid codes. White fonts on black boxes represent 100% similarity, white fonts on grey boxes denote <80% similarity, and black fonts on grey boxes show <60% similarity. Abbreviations: *Dps*SSB *Desulfotalea psychrophila* (NCBI Reference Sequence: WP_011189820.1), *Fps*SSB *Flavobacterium psychrophilum* (NCBI Reference Sequence: WP_011963776.1), *Par*SSB *Psychrobacter arcticus* (NCBI Reference Sequence: AAZ19531.1), *Pcr*SSB *Psychrobacter cryohalolentis* (NCBI Reference Sequence: ABE75735.1), *Pin*SSB *Psychromonas ingrahamii* (NCBI Reference Sequence: WP_011771629.1), *Ppr*SSB *Photobacterium profundum* (NCBI Reference Sequence: WP_011219846.1), *Pto*SSB *Psychroflexus torquis* (NCBI Reference Sequence: WP_015023871.1), *Swo*SSB *Shewanella woodyi* (NCBI Reference Sequence: WP_012323283.1), *Eco*SSB *Escherichia coli* K12 (NCBI Reference Sequence: YP_492202.1), *Bsu*SSB *Bacillus subtilis* (NCBI Reference Sequence: NP_391970.1), *Tte*SSB3 *Thermoanerobacter tengcongensis* MB4 (NCBI Reference Sequence: AAM25884.1), and *Tma*SSB *Thermotoga maritima* MSB8 (NCBI Reference Sequence: WP_004081225.1). An arrow indicates the boundary between the N-and C-terminal domains.

Figure 1 shows the multiple amino acid alignment of the SSB proteins from the psychrophilic bacteria under study, from *Shewanella woodyi* (GenBank accession No. NC_010506; [22]), mesophilic *E. coli* (GenBank Accession No. NC_007779; [23]) and *Bacillus subtilis (*GenBank Accession No. NC_000964; [24]), and from the thermophilic *Thermoanerobacter tengcongensis* (GenBank Accession No. NC_003869; [25]) and *Thermotoga maritima* (GenBank Accession No. NC_000853; [26]) microorganisms. The protein sequences of the proteins under scrutiny share a 26-70% identity and a 46-75% similarity with the *E. coli* K12 SSB, a 21-53% identity and 38-66% similarity with the *Shewanella woodyi* SSB, a 21-31% identity and 37-48% similarity with the *B. subtilis* SSB, a 21-36% identity and 36-53% similarity with the *Thermoanaerobacter tengcongensis* SSB3, and a 19-31% identity and 34-52% similarity with the *Thermotoga maritima* (Table 2). The similarity between these proteins refers primarily to the N-terminal domain and the four or five terminal amino acids of C-terminal domain which are common in all the known bacterial SSB proteins.

**Table 2:** **Identity and similarity of the SSB proteins under study to the *Eco*SSB, *Swo*SSB, *Bsu*SSB, *Tte*SSB3, and *Tma*SSB**

**SSB**	***Dps*SSB**	***Fps*SSB**	***Par*SSB**	***Pcr*SSB**	***Pin*SSB**	***Ppr*SSB**	***Pto*SSB**
Identity to *Eco*SSB	41%	26%	49%	45%	45%	70%	33%
Similarity to *Eco*SSB	56%	46%	57%	56%	58%	75%	49%
Identity to *Swo*SSB	34%	21%	47%	46%	53%	53%	30%
Similarity to *Swo*SSB	42%	38%	55%	55%	66%	63%	42%
Identity to *Bsu*SSB	28%	21%	31%	31%	28%	30%	21%
Similarity to *Bsu*SSB	46%	37%	47%	47%	40%	48%	38%
Identity to *Tte*SSB3	36%	29%	23%	22%	21%	22%	29%
Similarity to *Tte*SSB3	53%	42%	39%	39%	36%	40%	41%
Identity to *Tma*SSB	31%	25%	20%	21%	19%	25%	23%
Similarity to *Tma*SSB	52%	46%	34%	34%	34%	43%	41%

The C-terminal domain of bacterial SSBs contains a high number of negatively charged amino acid residues, which are required for the interaction with other proteins but are not essential to DNA binding. In the C-terminal domains of proteins under analysis in this study, the content of negatively charged residues is similar to, or even higher than, that found in the *Eco*SSB.

The *Eco*SSB base-stacking residues are Trp-40, Trp-54, Phe-60, and Trp-88. In contrast to the *Tma*SSB or *Tte*SSB3, the location of these residues is precisely preserved in the *Pin*SSB and *Ppr*SSB. In the *Fps*SSB and *Pto*SSB, this location is shifted with one amino acid residue, and instead of tryptophan, they have a tyrosine at position 39, and arginine residues rather than phenylalanine residue at position 59. The displacement of two amino acid residues is observed in the *Par*SSB and *Pcr*SSB, where the 86^th^ position is occupied by tyrosine and not by tryptophan. In the *Dps*SSB, the location of the base-stacking residues is shifted with four residues, namely Trp-36, and then with five; Trp-49, Trp-55, Trp-83, while tryptophan replaces phenylalanine in the 55^th^ position. With the exception of arginine, the amino acids residues thus replaced are also aromatic and, in participating in ssDNA binding, can play an analogous role to those residues in the *Eco*SSB. Highly conserved His-55, Gln-76 and Gln-110 residues, important for the homotetramerization of the *Eco*SSB, are present in the *Ppr*SSB protein. In the other proteins under study, only histidine residues were found, at the 55^th^ position in the *Pin*SSB, the 54^th^ position in the *Fps*SSB and *Pto*SSB, the 54^th^ position in the *Par*SSB and *Pcr*SSB, and the 50^th^ position in the *Dps*SSB.

### Oligomerization status

**Figure 2:**
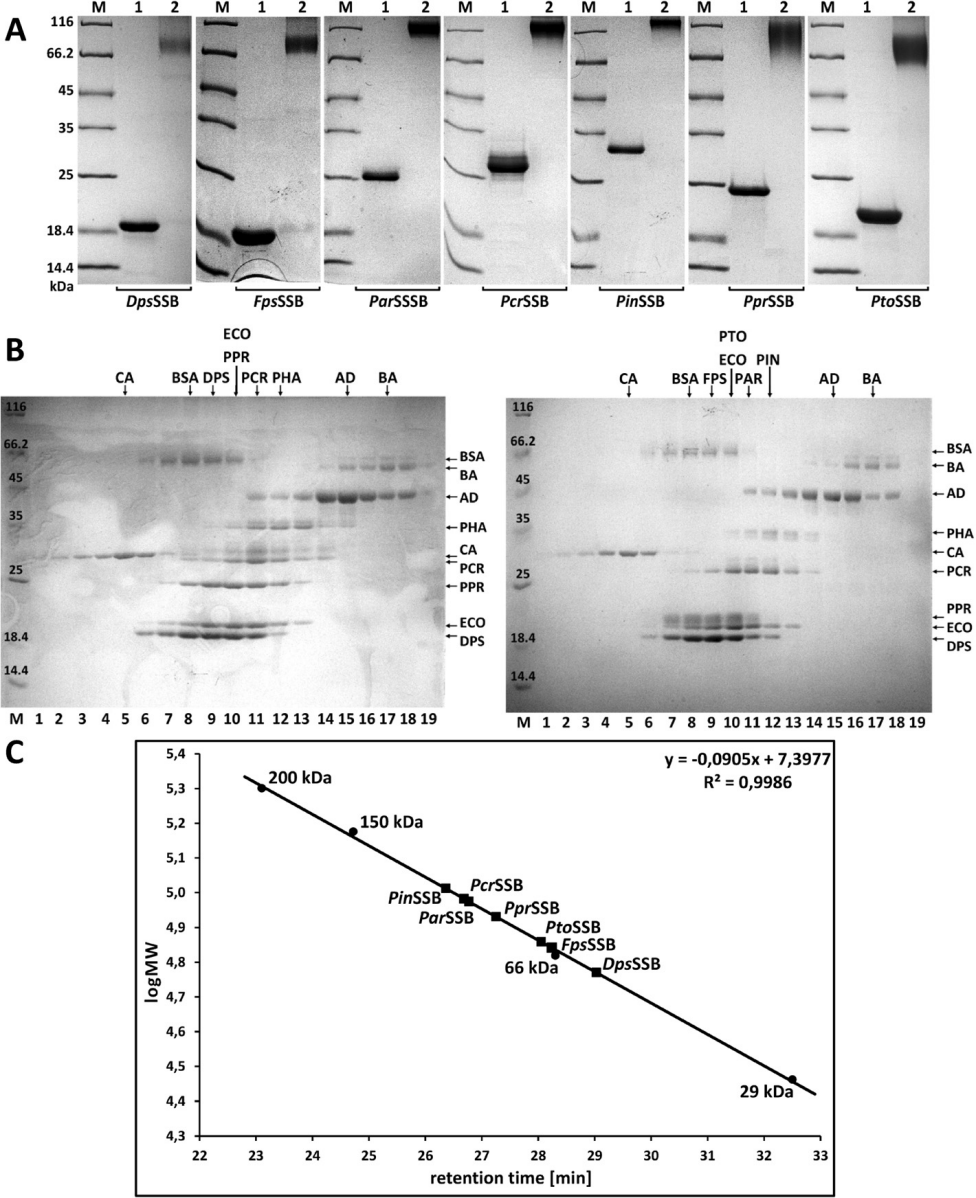
**Results of chemical cross-linking, ultracentrifugation and gel filtration experiments of SSB proteins. A**: The results of chemical cross-linking experiments using 0.5% (v/v) glutaraldehyde with the SSB proteins under study, for 15 min at 25°C (lanes 2) and non-cross-linked samples (lanes 1). The fractions were analyzed by SDS-PAGE. **B**: Sedimentation analysis of the psychrophilic SSB proteins, *Pha*SSB, *Eco*SSB and standard proteins. 50 μl of 300 μM SSBs and standard proteins were centrifuged in linear 15 to 30% (w/v) glycerol gradients, as described in the Methods section. Lane M: Unstained Protein Weight Marker (Fermentas, Lithuania), with the molecular mass of proteins marked. Lane 1–19: fraction number. The fractions with proteins were analyzed by SDS-PAGE. The fractions at which the maximal amount of protein appears are shown by arrows. The standard proteins used are CA, carbonic anhydrase (29 kDa); BSA, bovine serum albumin (66 kDa); AD, alcohol dehydrogenase (150 kDa), and BA, β-amylase (200 kDa). **C**: Analytical gel filtration of the psychrophilic SSB proteins under study. A standard linear regression curve is shown. It was generated by plotting the log of the molecular mass of the calibration proteins against their retention times [min]. The calibration proteins include β-amylase (200 kDa), alcohol dehydrogenase (150 kDa), bovine albumin (66 kDa) and carbonic anhydrase (29 kDa).

In chemical cross-linking experiments using glutaraldehyde, the *Dps*SSB, *Fps*SSB and *Pto*SSB complexes were found at a position corresponding to a molecular mass of approximately 80 kDa, the *Ppr*SSB complexes were found at a position corresponding to a molecular mass of about 100 kDa, the *Par*SSB and *Pcr*SSB complexes were found at a position corresponding to a molecular mass of around 116 kDa, and the *Pin*SSB complexes were found at a position corresponding to a molecular mass of approximately 140 kDa (Figure 2A). We observed that the psychrophilic SSB proteins in question have anomalous mobility in SDS-PAGE gels than would be expected on the basis of their predicted molecular masses. This phenomenon has also been observed in SSBs from *Shewanella* strains [27] and could be a characteristic feature of psychrophilic single-stranded DNA-binding proteins. The SSBs from *D. psychrophila*, *F. psychrophilum* and *P. torquis* were found at a position corresponding to a molecular mass of around 20 kDa (Figure 2A), while their calculated molecular masses are 15.6, 15.9 and 17.1 kDa, respectively. The *Ppr*SSB was found at a position corresponding to a molecular mass of approximately 25 kDa, while its calculated molecular mass is 20.4 kDa (Figure 2A). The *Par*SSB, *Pcr*SSB and *Pin*SSB were found at positions corresponding to molecular masses of around 25, 27 and 32 kDa, although their predicted molecular masses are 22.8, 23.3 and 25.1 kDa, accordingly (Figure 2A). Taken together, these results confirmed our prediction that the *Dps*SSB, *Fps*SSB, *Par*SSB, *Pcr*SSB, *Pin*SSB, *Ppr*SSB and *Pto*SSB exist as homotetramers in solution.

The oligomerization status of the SSBs was also analyzed by centrifugation in 15 to 30% (w/v) glycerol gradients. To prevent nonspecific aggregation of the proteins during the experiments, NaCl at a final concentration of 0.5 M was added to the solutions used for the gradients. The centrifugation in was carried out three times, and the same sedimentation behaviors were observed in all the independent tests. The sedimentation patterns of the SSB proteins in question, the *Pha*SSB, the *Eco*SSB and the standard proteins in the glycerol gradients suggest that all SSB proteins under study form homotetramers in the solution (Figure 2B).

An analytical gel filtration chromatography analysis of the purified psychrophilic SSBs revealed a single peak for each protein. As calculated using a regression curve equation, there was a peak with a molecular mass of 59 kDa for the *Dps*SSB, 69.5 kDa for the *Fps*SSB, 94.4 kDa for the *Par*SSB, 96.1 kDa for the *Pcr*SSB, 102.8 kDa for the *Pin*SSB, 85.4 kDa for the *Ppr*SSB, and 72.3 kDa for the *Pto*SSB, (Figure 2C). The native molecular mass of each peak represents 3.8 for the *Dps*SSB mass monomer, 4.4 for the *Fps*SSB mass monomer, 4.1 for the *Par*SSB, *Pcr*SSB and *Pin*SSB mass monomers, and 4.2 for the *Ppr*SSB and *Pto*SSB mass monomers, respectively. Psychrophilic single-stranded DNA binding proteins therefore exist in solution as homotetramers.

### ssDNA binding properties

**Figure 3:**
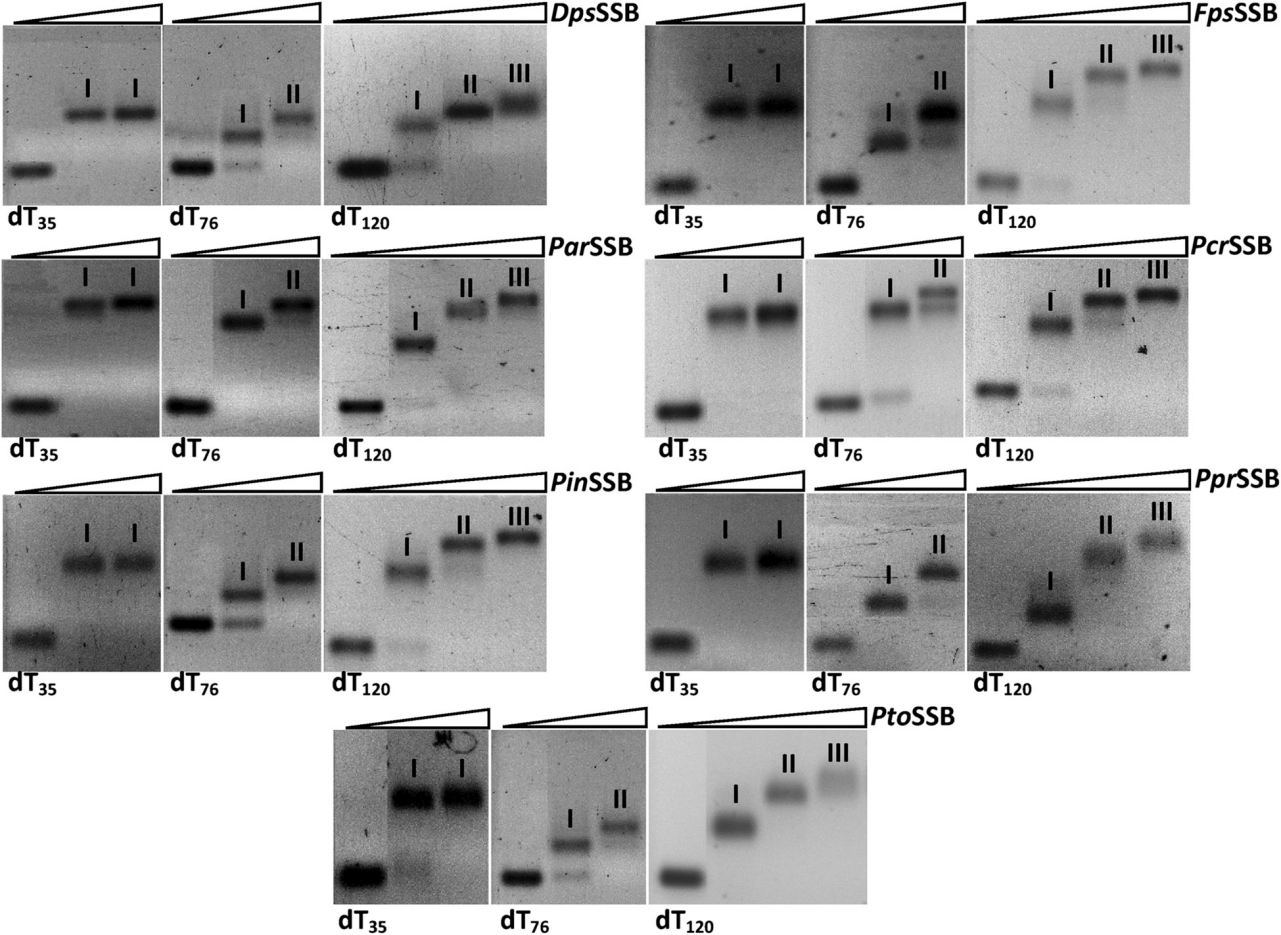
**Binding of SSB proteins to oligo (dT).** Fixed quantities (10 pmol) of 5′-end fluorescein-labelled oligonucleotides (dT)_35_, (dT)_76_ and (dT)_120_ were incubated with 50, 100 and 200 pmol of the SSB proteins in 20 μl reaction mixtures for 10 min at 25°C. Symbols I, II and III describe SSB:dT complexes.

The purified SSB proteins were analyzed for single-stranded DNA binding activity. In these experiments, a fixed concentration of (dT)_n_ (n = 35, 76 or 120 nucleotides in length) were incubated with various SSB concentrations and the resulting complexes were analyzed by agarose gel electrophoresis (Figure 3). When dT_35_ was incubated with increasing concentrations of each of the SSB proteins, a single band of reduced mobility was observed and remained constant even at a higher protein concentration (complex I). A band with the same mobility was observed for (dT)_76_ at a low protein concentration, but a second band with a lower mobility was observed at a high protein concentration (complex II). When SSB:dT_120_ complexes were analyzed, a third band with a lower mobility was detected (complex III). This implies that the length of ssDNA required for efficient protein binding is less than 35 nucleotides long.

**Figure 4:**
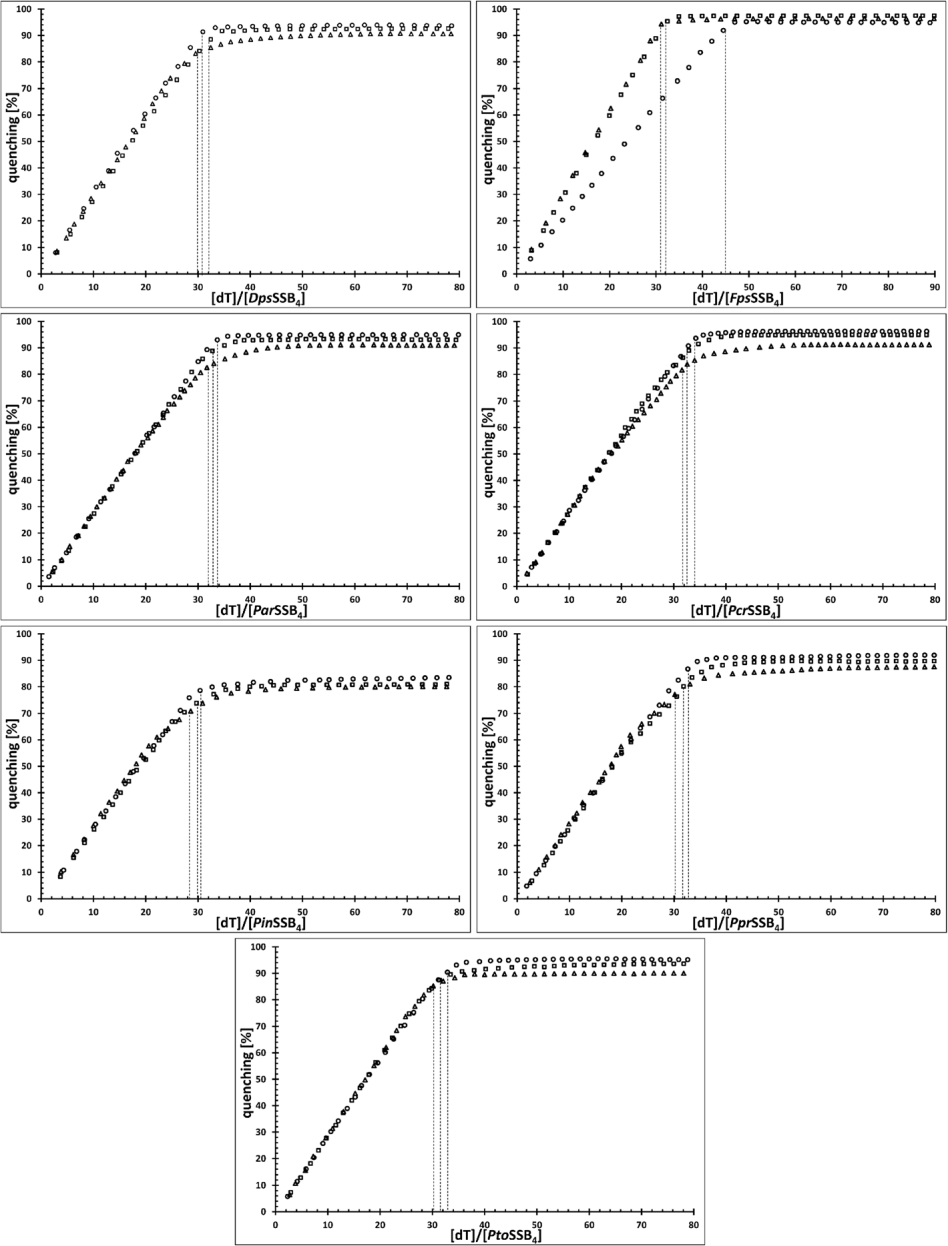
**Inverse fluorescence titration of SSB proteins with oligo(dT)**_**76**_**.** The 1.5 nmol samples of the SSB proteins under study were titrated with (dT)_76_ at 2 mM (Δ), 100 mM (□) and 300 mM (○) NaCl binding buffer.

In order to explore the binding properties of all the proteins in question further, we used fluorescence spectroscopy. All the bacterial SSBs which have been studied to date have shown a dramatic decrease of tryptophan fluorescence when binding to ssDNA. With an excitation wavelength of 295 nm, the emission spectrum of SSB proteins at 25°C reached its maximum at 348 nm, which is consistent with tryptophan fluorescence. On the addition of a saturating quantity of (dT)_76_, the intrinsic fluorescence at 348 nm was quenched by 93±3% for the *Dps*SSB, *Fps*SSB, *Par*SSB, *Pcr*SSB, and *Pto*SSB, by 90±3% for the *Ppr*SSB, and by 81±3% for the *Pin*SSB. It was salt independent. The estimated binding site was determined as being approximately 30 ± 2 nucleotides long for the *Pin*SSB, 31 ± 2 nucleotides for the *Dps*SSB and 32 ± 2 nucleotides for the *Par*SSB, *Pcr*SSB, *Ppr*SSB, and *Pto*SSB. Practically no binding mode transition was observed when changing the ionic strength from low to high salt (Figure 4). However, for the *Fps*SSB, a binding-mode transition of 31 ± 2 nucleotides at low salt concentrations and 45 ± 2 at high ones was observed.

### dsDNA melting point destabilization

**Figure 5:**
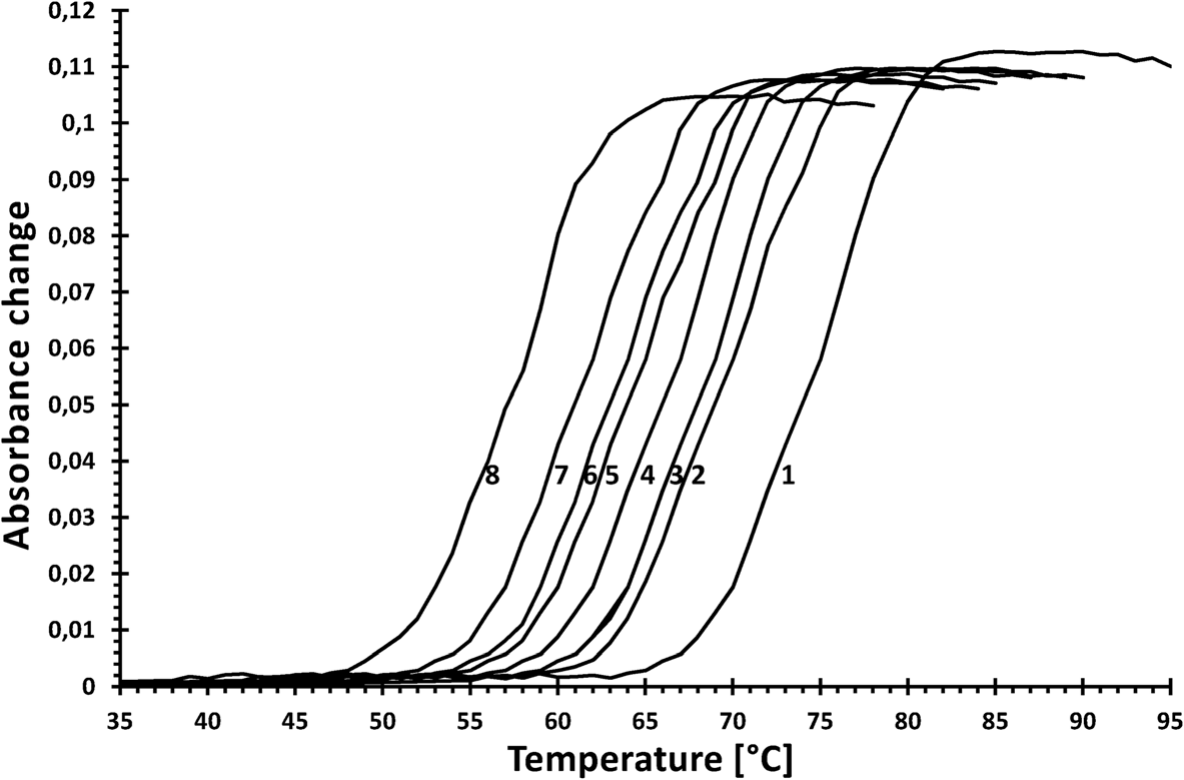
**Melting profiles of dsDNA and its complexes with SSB proteins.** A 0.67 nmol sample of duplex DNA (44 bp) was incubated alone (1) and with 4 nmol of the *Dps*SSB (2), *Fps*SSB and *Pin*SSB (3), *Par*SSB (4), *Ppr*SSB(5), *Pcr*SSB (6), *Eco*SSB (7) and *Pto*SSB (8), in a standard buffer containing 0.1 NaCl. Absorbance changes were measured at 260 nm.

A destabilization of DNA double strands in the presence of SSB must be expected as a thermodynamic consequence of SSB proteins binding specifically to ssDNA and not to dsDNA. The results of duplex DNA (44 bp) destabilization by the *Dps*SSB, *Fps*SSB, *Par*SSB, *Pcr*SSB, *Pin*SSB, *Ppr*SSB, *Pto*SSB, and *Eco*SSB are shown in Figure 5. The melting temperature of dsDNA in 0.1 M NaCl is decreased from 75 to 70°C by the *Dps*SSB, from 75 to 69°C by the *Fps*SSB and *Pin*SSB, from 75 to 67°C by the *Par*SSB, from 75 to 65°C by the *Ppr*SSB, from 75 to 64°C by the *Pcr*SSB, and from 75 to 58°C by the *Pto*SSB. In comparison, the melting temperature of the dsDNA is decreased from 75 to 62°C by the *Eco*SSB under the same conditions. The experiments were repeated three times with the same results on each occasion.

### Thermostability

**Figure 6:**
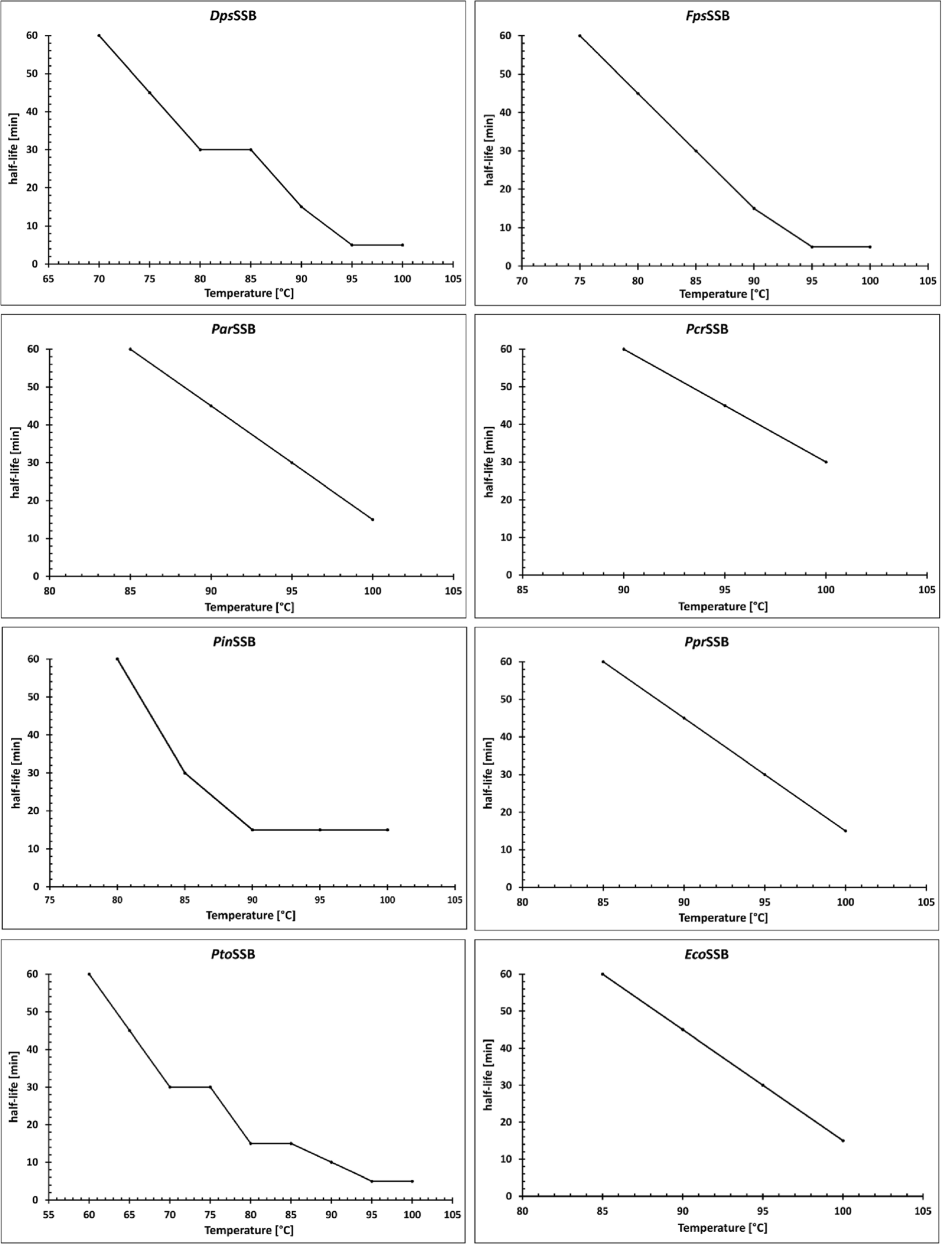
**The half-lives of the SSB proteins.** A fixed quantity of each SSB protein was incubated at temperatures ranging from 60°C to 100°C for 0, 1, 2.5, 5, 10, 15, 30, 45, and 60 min. 0.05 pmol 5′-end fluorescein-labelled oligonucleotide (dT)_35_ was then added. The protein-DNA complexes were separated from the free DNA by 2% agarose gel electrophoresis. The incubation periods for each temperature, where 50% of (dT)_35_ was bound, were noted.

The results of the indirect thermostability experiments are shown in Figure 6. Although the proteins come from psychrophilic bacteria, they have a high thermostability. The half-lives of the ssDNA-binding activities of the SSBs at 100°C and 95°C are 5 min for the *Dps*SSB, *Fps*SSB and *Pto*SSB, and 15 min for the *Pin*SSB. The thermostability of the *Par*SSB and *Ppr*SSB was 15 min at 100°C and 30 min at 95°C, while for the *Pcr*SSB, the half-lives were 30 and 45 min at those temperatures. The *Dps*SSB, *Fps*SSB and *Pin*SSB proteins share half-lives of 15 min at 90°C and 30 min at 85°C. A 50% loss of ssDNA-binding activity at 90°C was observed for the *Pto*SSB after 10 min of incubation, for the *Par*SSB and *Ppr*SSB after 45 min, and for the *Pcr*SSB after 60 min. The thermostability of the *P. torquis* SSB was 15 min at 85°C and 80°C, 30 min at 70°C, and 45 min at 65°C. There is a 50% decline in the activity of the *Par*SSB and *Ppr*SSB after 60 min at a temperature of 85°C and in that the *Dps*SSB, *Fps*SSB and *Pin*SSB after 30, 45 and 60 min at 80°C, respectively. A half-life of 60 min was observed for the *Fps*SSB at 75°C and for the *Dps*SSB and *Pto*SSB at 60°C. In comparison, under the same conditions, the activity of the *Eco*SSB decreased by 50% after 15 min at 100°C, 30 min at 95°C, 45 min at 90°C, and 60 min at 85°C.

**Figure 7:**
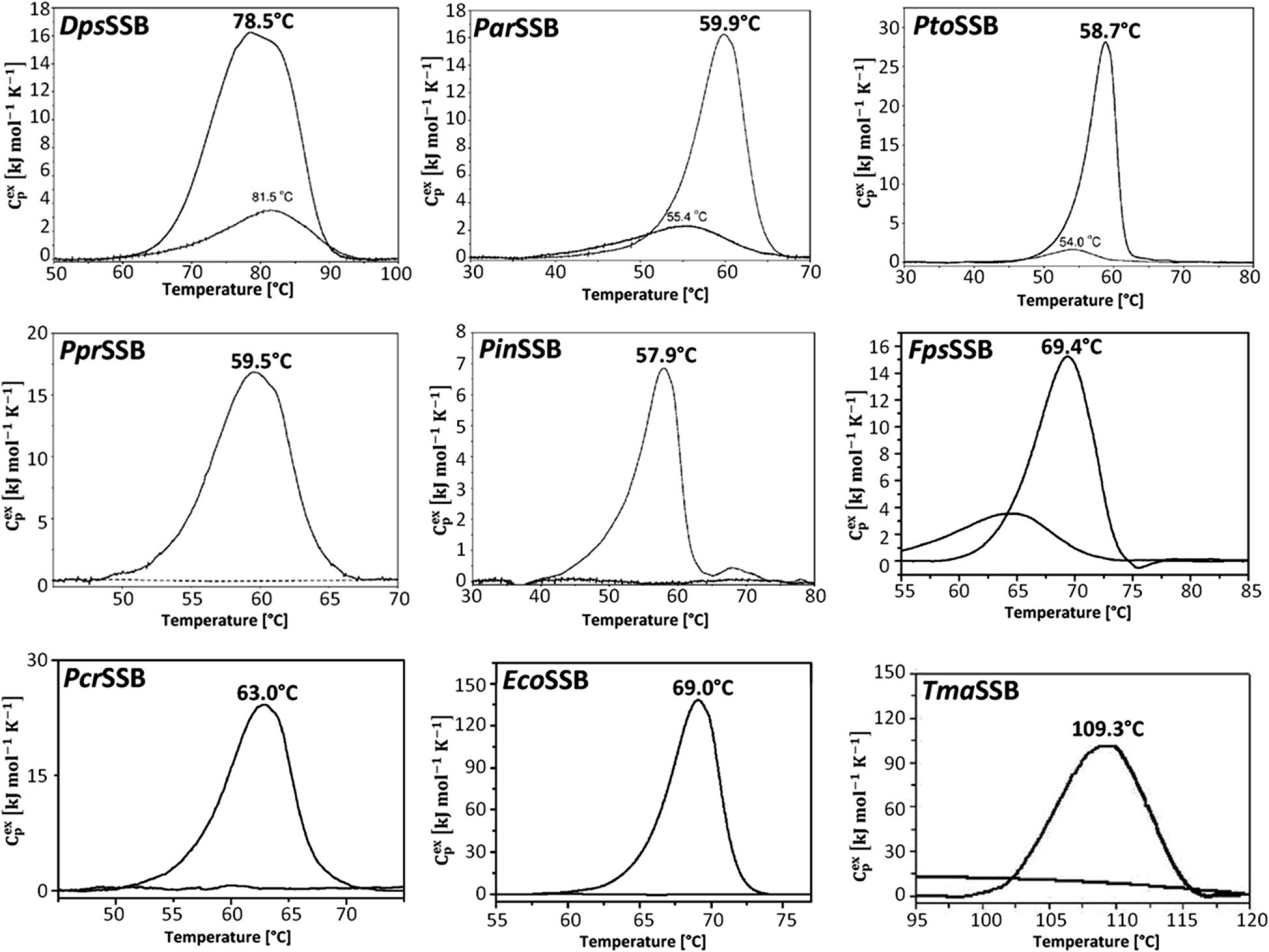
**DSC thermograms of SSB proteins.** Samples containing 2 mg/ml of the *Dps*SSB, *Par*SSB, *Pto*SSB, *Ppr*SSB, *Pin*SSB, *Fps*SSB, *Pcr*SSB, *Eco*SSB, and *Tma*SSB were analyzed in 50 mM of potassium phosphate buffer pH 7.5 and 150 mM NaCl. The melting temperatures are shown.

When analyzed by differential scanning microcalorimetry (DSC), the thermal unfolding was found to be an irreversible process in the *Pcr*SSB, *Pin*SSB and *Ppr*SSB, and partially reversible for the *Dps*SSB, *Fps*SSB, *Par*SSB and *Pto*SSB, as can be seen in the rescan thermograms (Figure 7). At melting temperatures (T_m_) of 59.9°C, 63°C, 57.9°C, 59.5°C, and 58.7°C, respectively, the *Par*SSB, *Pcr*SSB, *Pin*SSB, *Ppr*SSB, and *Pto*SSB had a lower thermostability than the *Eco*SSB which had a T_m_ of 69.0°C. The *Dps*SSB and *Fps*SSB, with T_m_ of 78.5°C and 69.4°C, demonstrated more thermostablity than the *Eco*SSB, but still had less thermostable than the *Tma*SSB, at a T_m_ 109.3°C [28]. The thermograms of these SSB proteins showed no characteristic signs of heavily aggregated proteins after heat denaturation. Although the proteins under study come from psychrophilic microorganisms, they have a relatively high thermostability.

## Discussion

In this report, we have described the purification and characterization of single-strand DNA-binding proteins from obligate psychrophilic bacteria *D. psychrophila*, *P. ingrahamii*, *P. profundum* and *P. torquis* and the facultative psychrophilic bacteria *F. psychrophilum*, *P. arcticus* and *P. cryohalolentis*. All the proteins investigated form tetramers in solution, as demonstrated by three methods: chemical cross-linking experiments, sedimentation analysis and gel filtration chromatography. The results of the sequence analysis verified that an ssDNA binding domain in one monomer of each protein possesses a canonical oligonucleotide binding fold (OB-fold) very similar to that observed in the structure of the *E. coli* SSB. The OB-fold in the proteins in question demonstrated a high level of identity and similarity to *Eco*SSB, with *Dps*SSB at 55% and 75%, *Fps*SSB at 38% and 52%, *Par*SSB at 57% and 73%, *Pcr*SSB at 58% and 74%, *Pin*SSB at 61% and 82%, *Ppr*SSB at 82% and 90%, and *Pto*SSB at 42% and 62%, which was somewhat surprising, given that they come from taxonomical distant microorganisms living in different environments. They show a high differential in both the molecular mass of their monomers and the length of their amino acid sequences. Of the known SSBs with one OB-fold, the *Dps*SSB is the smallest and the *Fps*SSB is the shortest.

The *Par*SSB, *Pcr*SSB, *Pin*SSB, *Ppr*SSB and *Pto*SSB have melting temperatures (T_m_) of 59.9°C, 63°C, 57.9°C, 59.5°C and 58.7°C, respectively, which are somewhat lower than for the *Eco*SSB, at 69.0°C. With T_m_ of 78.5°C and 69.4°C, the *Dps*SSB and *Fps*SSB are more thermostable than the *Eco*SSB, but their thermostability is not at the level of that for the thermophilic *Tma*SSB, with a T_m_ 109.3°C, or even for the *Taq*SSB, with T_m_ of 86.8°C [28]. The indirect thermal stability tests showed that both mesophilic and psychrophilic SSBs retain their binding activity at temperatures higher than their melting temperature for specified incubation times. These proteins could thus be used in molecular biology in high-temperature reactions such as nucleic acid amplification.

It is well known that cold-adapted proteins exhibit greater flexibility than their mesophilic counterparts, with a reduced number of weak interactions. This flexibility is often associated with the reduced stability of the psychrophilic protein. In comparison to their mesophilic equivalents, these proteins also often feature a higher Gly content; a lower basic amino acid content, particularly Arg, with a decreased Arg/(Arg + Lys)ratio; a lower Pro content, resulting from Pro deletion or substitution by other small residues such as Ala, for example; fewer hydrogen bonds and aromatic interactions; and residues which are more polar, and less hydrophobic, resulting in the destabilization of the hydrophobic core. All these characteristics work together to increase the number of degrees of conformational freedom by introducing flexible residues on the protein surface and destabilizing the protein core by weakening the intermolecular forces. In this context, the *Dps*SSB, *Fps*SSB, *Par*SSB, *Pcr*SSB, *Pin*SSB, *Ppr*SSB, and *Pto*SSB proteins have some cold adaptation qualities.

With the exception of the *Pcr*SSB and *Ppr*SSB, the proteins under study have a charged residues content of Asp, Glu, Lys, His and Arg, with *Dps*SSB at 24.5%, *Fps*SSB at 29.3%, *Par*SSB at 20.1%, *Pcr*SSB at 18.3%, *Pin*SSB at 21.2%, *Ppr*SSB at 18.0%, and *Pto*SSB at 30.4%) which is higher than the SSB from *E. coli*, at 19.7% (Table 3). Furthermore, the *Fps*SSB and *Pto*SSB share a charged amino acid residues content which is close to that of the *Tte*SSB3, at 30.7%. In the thermophilic proteins, these residues may be involved in the ionic networks stabilization of the interdomain surface. In the *Dps*SSB, *Fps*SSB, *Par*SSB, *Pcr*SSB, *Pin*SSB, *Ppr*SSB and *Pto*SSB, the content of Arg residues and the Arg/(Arg + Lys) ratio are 7.0% and 0.63, 2.9% and 0.22, 4.7% and 0.53, 4.6% and 0.55, 4.5% and 0.43, 4.4% and 0.54, and 2.6% and 0.20, respectively. These factors are definitely lower in the psychrophilic SSBs than in their mesophilic *E. coli* equivalent, at 5.6% and 0.62, with the exception of *Dps*SSB, and the thermophilic SSBs *Tte*SSB3, at 6.0% and 0.53, and *Tma*SSB, at 10.6% and 0.75). This feature has been considered as a hallmark of psychrozymes [29-35]. The ability to form multiple salt bridges with acidic Asp and/or Glu amino acid residues and hydrogen bonds with other amino acids is normal for arginine. The decrease of Arg content, even the conservative replacement of Arg with Lys, entails a reduction in the number of salt bridges.

**Table 3:** Percentage amino acid content of the SSB proteins under comparison

**SSB**	**Ala**	**Ile**	**Leu**	**Val**	**Met**	**Gly**	**Pro**	**Lys**	**Arg**	**Asp**	**Glu**	**Gln**	**Asn**	**Ser**	**Thr**	**His**	**Trp**	**Phe**	**Tyr**	**Cys**
*Dps*SSB	7.0	6.3	4.9	3.5	2.8	11.3	4.2	4.2	7.0	4.9	7.7	4.9	6.3	9.2	7.0	0.7	2.8	1.4	2.8	0.7
*Fps*SSB	4.3	7.9	5.0	6.4	2.1	6.4	2.1	10.0	2.9	5.0	9.3	2.1	7.1	8.0	10.7	2.1	1.4	4.3	3.6	1.4
*Par*SSB	8.0	5.2	3.3	2.8	1.9	16.4	4.7	4.2	4.7	5.6	4.2	12.2	8.0	5.6	4.2	1.4	0.9	3.3	3.3	0
*Pcr*SSB	6.8	4.6	2.7	2.7	1.8	16.9	4.6	3.7	4.6	5.0	4.1	12.8	10.0	7.3	4.1	0.9	0.9	3.2	3.2	0
*Pin*SSB	7.7	1.8	3.6	4.5	3.6	6.8	9.9	5.9	4.5	4.5	5.4	17.6	6.3	3.6	6.3	0.9	1.8	2.3	2.7	0.5
*Ppr*SSB	7.7	3.3	3.8	6.6	2.7	10.4	7.1	3.8	4.4	3.8	5.5	21.3	4.4	3.8	3.8	0.5	2.2	2.2	2.7	0
*Pto*SSB	5.3	5.3	4.6	6.0	2.6	6.0	7.3	10.6	2.6	5.3	9.9	5.3	4.6	3.3	9.3	2.0	1.3	3.3	3.3	2.0
*Eco*SSB	7.3	2.8	4.5	7.3	3.4	16.3	6.7	3.4	5.6	4.5	5.6	10.1	4.5	5.6	5.0	0.6	2.2	2.2	2.2	0
*Tte*SSB3	4.0	5.3	7.3	8.7	2.0	6.0	6.0	5.3	6.0	10.7	8.0	1.3	4.0	6.7	8.0	0.7	2.0	6.0	1.3	0
*Tma*SSB	5.0	4.3	5.7	9.2	2.8	4.3	7.1	3.5	10.6	6.4	12.8	0.7	2.1	5.0	10.6	0	0.7	7.8	1.4	0

The glycine content in psychrophilic SSBs, particularly in the *Dps*SSB, at 11.3%, *Par*SSB, at 16.4%, *Pcr*SSB, at 16.9%, and *Ppr*SSB, at 10.4%, and in the mesophilic *Eco*SSB, at 16.3%, is much higher than in the thermophilic SSBs, at 6.0% and 4.3% for *Tte*SSB3 and *Tma*SSB, respectively. This accords with the known tendency of thermostable proteins to have a preference for a decrease in the Gly content in positions of low structural importance for fold conservation [36,37].

The high content of glutamine and asparagine residues observed in the *Par*SSB, at 20.0%, *Pcr*SSB, at 23.0%, *Pin*SSB, at 24.93, and *Ppr*SSB, at 25.4% is one and a half times greater than that of the *Eco*SSB, at 14.5% and much higher than for the thermophilic SSBs, at 5.3% and 2.8% for the *Tte*SSB3 and *Tma*SSB, respectively. Of the 39 glutamine residues in the *Pin*SSB and *Ppr*SSB, 34 are located in the C-terminal fragment of the former and 29 in that of the latter, which represents, respectively, 30.4% and 38.2% of that domain. At up to 9 rests side by side, the glutamine residue repetitions in the C-terminal fragment of the *Ppr*SSB are extremely numerous, endowing the domain with a highly hydrophilic character. This area is reminiscent of the ‘glutamine-rich (Q-rich) regions’ in proteins other than SSBs, which form a ‘polar zipper’ and with which different protein subunits interact in a specific manner.

The ratio of polar to non-polar amino acid residues is one of the major determinants of protein stability and increasing the fraction of polar and charged residues leads to protein disorder [29]. The content of polar amino acid residues N, Q, S, T, and Y in the *Dps*SSB, *Fps*SSB, *Par*SSB, *Pcr*SSB, *Pin*SSB, *Ppr*SSB, and *Pto*SSB is 30.2%, 31.5%, 33.3%, 37.4%, 36.5%, 36.0% and 25.8%, respectively. With the exception of *Pto*SSB, this is considerably more than that found in the mesophilic *Eco*SSB, at 27.4%, and very much more than that found in the thermophilic SSBs, at 21.3% and 19.8% for *Tte*SSB3 and *Tma*SSB, accordingly. Russell [35] and Zuber [38] noticed that psychrophilic proteins appear to have more polar residues than thermophiles or mesophiles do, which is consistent with our research. *A*s mentioned previously, a lower Pro content is one of the features of cold-adapted proteins. In the proteins under study, only the *Dps*SSB, at 4.2%, *Fps*SSB, at 2.1%, *Par*SSB, at 4.7%, and *Pcr*SSB, at 4.6%, possess a lower proline content than their mesophilic and thermophilic counterparts, with *Eco*SSB, at 6.7%, *Tte*SSB3, at 6.0% and *Tma*SSB, at 7.1%.

Tiny and small amino acids were observed to be significantly increased in the beta sheets and loops of the psychrophilic proteins as compared with their mesophilic counterparts [39]. Their compositions in the SSBs in question are less than in the *Eco*SSB, at 61.0%. Moreover, the *Fps*SSB and *Pin*SSB have a lower content of these residues, at 54%, than the *Tte*SSB3, at 56%. The composition of the small and tiny residues in the *Ppr*SSB, at 50%, and the *Pto*SSB, at 52%, is even less than in the *Tma*SSB, at 53%.

Aromatic amino acid residues are known to play an important role in stabilizing the three-dimensional structure of proteins. Psychrophilic proteins usually display a decrease in these amino acids. The psychrophilic SSBs deviate from this rule; all of proteins investigated show a higher content of these residues than the *Eco*SSB, at 6.6%. The *Fps*SSB has the same number of aromatic amino acids in its sequence as the *Tte*SSB3, namely 9.3%.

It was also observed that, in psychrophilic proteins, the number of hydrophobic amino acids is lower than for their mesophilic counterparts. The content of hydrophobic amino acid residues in the *Dps*SSB, *Fps*SSB, *Par*SSB, *Pcr*SSB, *Pin*SSB, *Ppr*SSB, and *Pto*SSB is 44.2%, 39.9%, 46.5%, 44.2%, 42.0%, 46.0% and 41.7%, respectively. The number of these residues in the psychrophilic SSB proteins is less than in the *Eco*SSB, at 52.7%. Moreover, the aromatic residue content in the *Par*SSB and *Ppr*SSB is close to that of the *Tma*SSB, at 46.9%.

Analysis of the amino acid sequence of the *Dps*SSB, *Fps*SSB, *Pin*SSB and *Pto*SSB shows the presence of cystein residues to a number of 1, 2, 1, and 3, respectively. To date, these amino acid residues have not been found in any known SSBs. A residue such as proline or cystein has a significant impact on the stability and rigidity of the conformational structure of proteins. The presence of cystein residues in psychrophilic SSBs may affect their stability, particularly if disulphide bridges are formed.

Single strand DNA binding proteins have the property of causing the destabilization of duplex DNA and the same is true of the psychrophilic SSBs under study. The greatest decrease in dsDNA melting temperature was observed in the presence of the *Pto*SSB, at 17°C, which was a more substantial change than in the presence of the *Eco*SSB, *Taq*SSB or *Tth*SSB, at 13°C in each case [40-42].

Studies of other SSBs have often shown that the size of the binding site depends on the salt concentration. At least two distinctly different DNA-binding modes have been described for the *Eco*SSB, for example [3]. In high salt concentrations, 65 nucleotides bind per *Eco*SSB tetramer, with a fluorescence quench of almost 90% whereas, in low salt concentrations, 35 nucleotides are sufficient to saturate the protein and quench its fluorescence by only 53%. Our current study has demonstrated that the binding site size of the *Dps*SSB, *Par*SSB, *Pcr*SSB, *Pin*SSB, *Ppr*SSB and *Pto*SSB has a constant value of approximately 30–32 nucleotides per tetramer, with one, salt-independent, DNA-binding mode. Binding-mode transition was only observed for the *Fps*SSB, at 31 ± 2 nucleotides at low salt concentrations and 45 ± 2 nucleotides at high ones. This is similar to the recently described psychrophilic *Pha*SSB, with 34 nucleotides per tetramer under low-salt conditions and 54–64 nucleotides at higher ones. This suggests that the *Fps*SSB and *Pha*SSB undergo a transition between ssDNA binding modes, something which is observed for the *Eco*SSB.

## Conclusion

The results showed that SSB proteins from psychrophilic microorganisms are typical bacterial SSBs and possess relatively high thermostability, offering an attractive alternative to other thermostable SSBs in molecular biology applications.

## Methods

### Bacterial strains, plasmids, enzymes and reagents

*D. psychrophila* LSv54 (DSM 12343), *P. arcticus* 273–4 (DSM 17307), *P. cryohalolentis* K5 (DSM 17306) and *P. ingrahamii* 37 (DSM 17664) were purchased from The Leibniz Institute DSMZ (German Collection of Microorganisms and Cell Cultures, Germany). *F. psychrophilum* JIP02/86 (LMG 13180), *P. profundum* (LMG 19446) and *P. torquis* ATCC 700755 (LMG 21429) were purchased from BCCM/LMG (The Belgian Co-ordinated Collections of Micro-organisms, Belgium). Genomic sequences for those strains are available and were published: *D. psychrophila* (GenBank accession no. NC_006138; [16]), *F. psychrophilum* (GenBank accession no. NC_009613; [17]), *P. arcticus* (GenBank accession no. NC_007204; [18]), *P. cryohalolentis* (GenBank accession no. NC_007969; Gene Bank Project: PRJNA58373), *P. ingrahamii* (GenBank accession no. NC_008709; [19]), *P. profundum* (GenBank accession no. NC_006370; [20]) and *P. torquis* (GenBank accession no. NC_018721; [15]).

The *E. coli* TOP10 (Invitrogen, USA) was used for genetic constructions and gene expression. The pBAD/*myc*-HisA plasmid (Invitrogen, USA) was used for constructing the expression system. The reagents for PCR were obtained from Blirt SA - DNA-Gdańsk (Poland). Specific primers, oligodeoxynucleotides and the oligonucleotides 5′-end-labelled with fluorescein were purchased from Sigma (USA). The restriction enzymes were purchased from NEB (USA). *Eco*SSB, *Pha*SSB and *Tma*SSB were produced and purified in our laboratory according to published procedure ( [7,28,43], respectively).

### Cloning of the *ssb*-like genes from psychrophilic bacteria

DNA from *D. psychrophila*, *F. psychrophilum*, *P. arcticus*, *P. cryohalolentis*, *P. ingrahamii*, *P. profundum* and *P. torquis* was isolated using an ExtractMe DNA Bacteria Kit (Blirt SA - DNA-Gdańsk, Poland). The specific primers for PCR amplification were designed and synthesized on the basis of the known *ssb*-like gene sequences. The forward (containing a *Nco*I recognition site) and reverse (containing a *Bgl*II or *Hin*dIII recognition site) primers are shown in Table 4. The boldface parts of primers sequences are complementary to the nucleotide sequences of the *ssb*-like genes, whereas 5′ overhanging ends of primers contain recognition sites for restriction endonucleases (underlined) and are designed to facilitate cloning. The PCR reaction solution (25 μl) consisted of: 0.2 μg of genomic DNA, 0.4 μM of each primer, 1 mM dNTPs, 2 mM MgCl_2_, 20 mM Tris–HCl, pH 8.8, 50 mM KCl, 10 mM (NH_4_)_2_SO_4_, 0.1% Triton X-100 and 2U *Pwo* DNA polymerase (Blirt SA DNA-Gdańsk, Poland). 35 cycles were performed, using the Veriti® 96 Well Thermal Cycler (Applied Biosystems, USA), with a temperature profile of 1 min at 94°C, 1 min at 60°C and 1 min at 72°C. The amplification products were analyzed by electrophoresis on 1% agarose gel stained with ethidium bromide, at a final concentration of 0.5 μg/ml. Specific PCR products were obtained and purified using the ExtractMe Gel-Out Kit (Blirt SA DNA-Gdańsk, Poland). The PCR products were digested with *Nco*I and *Bgl*II or *Hin*dIII (NEB, USA), then purified, using the ExtractMe Clean-Up Kit (Blirt SA DNA-Gdańsk, Poland) and ligated into pBAD/*myc*-HisA plasmid (Invitrogen, USA) between the *Nco*I and *Bgl*II or *Nco*I and *Hin*dIII sites. The *E. coli* TOP10 cells were transformed with the ligation mixtures and transformants were examined for the presence of the *ssb*-like genes, using a gel retardation assay and restriction analysis. One clone was selected and sequenced to confirm the presence of the *ssb*-like genes. The appropriate pBAD*Dps*SSB, pBAD*Fps*SSB, pBAD*Par*SSB, pBAD*Pcr*SSB, pBAD*Pin*SSB, pBAD*Ppr*SSB, and pBAD*Pto*SSB recombinant plasmids were obtained.

**Table 4:** The specific primers for PCR amplification

**Name**	**Primer sequence**
fpsssbNcoI	5′ GGA GGA C **CA TGG** GG**A ACG GAA CGT TAA ATA AAG TCA TG** 3′
fpsssbHindIII	5′ TTA AAG CTT**TTA AAA AGG CAA ATC ATT TTC TAC AG** 3′
pcrssbNcoI	5′ TTA CC **A TGG** GG**C GCG GTG TTA ATA AAG TTA TCA TC** 3′
pcrssbHindIII	5′ TTA AAG CTT**TCA GAA CGG AAT GTC ATC GTC** 3′
ptossbNcoI	5′ GGA GGA CC **A TGG** **CAG GAA CAC TCA ATA AAG TTA TGC** 3′
ptossbHindIII	5′ TTA AAG CTT**TTA AAA GGG TAG ATC ATC TTC CTC** 3′
pprssbNcoI	5′ GGA GGA CC **A TGG** **CCA GTC GTG GTG TAA ATA AGG** 3′
pprssbBglII	5′ TTA AGA TCT**CTA GAA TGG GAT ATC ATC ATC AAA ATC** 3′
dpsssbNcoI	5′ TTA CC **A TGG** **GGA TAA ATA AGG CAA TTT TAA TTG GTA ATC TAG** 3′
dpsssbHindIII	5′ TTA AAG CTT**CTA GAA GGG TAC GTC GTT AC** 3′
parssbNcoI	5′ GGA GGA CC **A TGG** **GGC GCG GTG TTA ATA AAG TTA TCA TC** 3′
parssbBglII	5′ TTA AGA TCT**CTA GAA AGG AAT GTC ATC GTC** 3′
pinssbNcoI	5′ TTA CC **A TGG** GG**T TTA ACC GAA GCG TAA ACA AAG TAG** 3′
pinssbHindIII	5′ TTA AAG CTT**CTA AAA AGG AAT ATC ATC ATC GAA ATC** 3′

The boldface parts of the primers sequences are complementary to the nucleotide sequences of the *ssb*-like genes and the underlined parts are the recognition sites for restriction endonucleases.

### Expression and purification of SSBs

The *E. coli* TOP10 strain transformed with pBAD*Dps*SSB, pBAD*Fps*SSB, pBAD*Par*SSB, pBAD*Pcr*SSB, pBAD*Pin*SSB, pBAD*Ppr*SSB or pBAD*Pto*SSB was grown at 30°C in Luria-Bertani medium, supplemented with 100 μg/ml of ampicillin, to an OD_600_ of 0.4, and was induced by incubation in the presence of arabinose, at a final concentration of 0.02%, for 20 h. The cells were then harvested by centrifugation at 4,612 × *g* for 20 min and the pellets were resuspended in 50 ml of buffer A (20 mM Tris–HCl pH 8.0, 50 mM NaCl, 1 mM EDTA pH 8.0, 0.1% Triton X-100). The samples were sonicated eight times, for 30 s at 4°C, and centrifuged at 10,000 × *g* for 25 min. The clarified supernatant was applied further directly onto QAE-cellulose column (50 ml bed volume, EMD, USA) preequilibrated with 4 vol buffer B (20 mM Tris–HCl pH 8.0, 50 mM NaCl, 1 mM EDTA pH 8.0). Each of SSB proteins was eluted with linear gradient of 0.05-2 M NaCl in buffer B. The SSB-containing fractions were detected by SDS-PAGE electrophoresis, after which, they were combined and loaded onto a ssDNA-cellulose column (5 ml, USB, USA) equilibrated with buffer C (20 mM Tris–HCl pH 8.0, 0.25 M NaCl, 1 mM EDTA pH 8.0). SSB proteins were eluted with 1.5 M NaCl and 50% ethylene glycol. The elution fractions were dialyzed against D buffer (20 mM Tris–HCl pH 8.0, 0.15 M NaCl) and concentrated to 2 mg/ml, using the Amicon Ultra-15 Filter Device MWCO 10000 (Millipore, USA). The purity of the SSBs was estimated using SDS-PAGE and the amounts were examined spectrophotometrically. The *E. coli* overexpression systems used in this study produced approximately 20 mg of purified SSB proteins from 1 L of induced culture. The purity of the protein preparations was 95-98%.

### Estimation of the native molecular mass

The native molecular mass of examined SSBs was determined by three independent methods: (i) chemical cross-linking, (ii) sedimentation in glycerol gradient and (iii) analytical gel filtration.

Chemical cross-linking experiments were carried out using 0.5% (v/v) glutaraldehyde for 15 min, with SSBs amount of 10 (*Par*SSB, *Pin*SSB), 50 (*Dps*SSB, *Pcr*SSB, *Ppr*SSB) or 100 (*Fps*SSB, *Pto*SSB) pmol, at 25°C. The reaction was quenched by the addition of 1 M Tris–HCl (pH 8.0), and the cross-linked protein solutions were then analyzed using SDS-PAGE (12%).

Linear 15 to 30% (w/v) glycerol gradients, containing loading buffer (50 mM Tris–HCl, pH 7.5, 0.5 M NaCl, 1 mM EDTA and 5 mM β-mercaptoethanol) were prepared in 5 ml Beckman centrifuge tubes. Standard proteins were: carbonic anhydrase (29 kDa), bovine albumin (66 kDa), alcohol dehydrogenase (150 kDa) and β-amylase (200 kDa) taken from Sigma Gel Filtration Markers Kit (Cat no. MWGF1000). 50 μl of a 300 μM *Dps*SSB, *Fps*SSB, *Par*SSB, *Pcr*SSB, *Pin*SSB, *Ppr*SSB and *Pto*SSB proteins in loading buffer, and the corresponding amounts of *Eco*SSB, *Pha*SSB and standard proteins, were layered over 3.5 ml of the glycerol gradient and were centrifuged in individual tubes. The gradients were centrifuged at 4°C in a Beckman SW 60 rotor at 46,000 rpm for 24 h; fractions were collected from the top. The proteins present in fractions were separated by SDS-PAGE.

Analytical gel filtration was carried out on a Superdex 200 HR75 10/300 GL column (Amersham Biosciences, USA), equilibrated with 20 mM Tris–HCl pH 7.5, 150 mM NaCl and 10 mM EDTA. The samples were eluted with the same buffer at a flow rate of 0.5 ml/min. The elution profile was monitored by recording the absorbance at 280 nm. The molecular weight of SSB proteins were determined by comparing the elution patterns with those of standard proteins, taken from Gel Filtration Markers Kit (Sigma, USA), including β-amylase (200 kDa), alcohol dehydrogenase (150 kDa), bovine albumin (66 kDa) and carbonic anhydrase (29 kDa).

### Agarose gel electrophoresis mobility shift assays (EMSA)

A fixed quantity (10 pmol) of 5′-end fluorescein-labelled oligonucleotides (dT)_35_, (dT)_76_ and (dT)_120_ were incubated with 50, 100 and 200 pmol of examined SSB proteins for 10 min at 25°C in a binding buffer (20 mM Tris–HCl pH 8.0, 100 mM NaCl and 1 mM EDTA) to a final reaction volume of 20 μl. Subsequently the reaction products with oligos were loaded onto 2% agarose gel without ethidium bromide and separated by electrophoresis in a TAE buffer (40 mM Tris acetate pH 7.5 and 1 mM EDTA). The bands corresponding to the unbound ssDNA and various SSB-ssDNA complexes were visualized under UV light and photographed.

### Fluorescence titration

Fluorescence titrations were carried out in a Perkin-Elmer LS-5B luminescence spectrometer as described earlier [44]. The binding reactions were assembled in 2 ml buffer of 20 mM Tris–HCl pH 8.0, 1 mM EDTA containing 2 mM, 100 mM or 300 mM NaCl and incubated at 25°C. A fixed quantity (1.5 nmol) of examined SSB proteins were incubated in the appropriate buffer at 25°C with increasing quantities of (dT)_76_ oligonucleotide at excitation and emission wavelengths of 295 and 348 nm, respectively. Binding curve analyses were carried out using Schwarz and Watanabe’s model [45].

### Melting point destabilization of dsDNA

Melting point curves were obtained by measuring the change in A_260_ in a Cary300Bio UV-Visible spectrophotometer (Varian) in 20 mM sodium phosphate buffer pH 7.5 containing 0.1 M NaCl and 1 mM EDTA [46]. A mixture of 0.67 nmol dsDNA and 4 nmol of particular SSB were gradually heated from 25°C to 95°C with heating rate of 1°C/min. The assay was performed using duplex DNA (44 bp) composed of two oligonucleotides: 5′-GAA CCG GAG GAA TGA TGA TGA TGA TGG TGC GGT TTG TCG GAC GG-3′ and 5′-CCG TCC GAC AAA CCG CAC CAT CAT CAT CAT CAT TCC TCC GGT TC-3′.

### Thermostability

The thermostability of the SSB proteins was determined by direct (DSC) and indirect methods.

Microcalorimetric measurements were performed using a NanoDSC microcalorimeter (Calorimetry Science Corporation, USA). Samples containing approximately 2.0 mg/ml SSB, in 50 mM of potassium phosphate buffer pH 7.5 and 150 mM NaCl were analyzed. The calorimetric scans were carried out between 0 and 100°C, with a scan rate of 1°C/min. The reversibility of the transition was checked by cooling and reheating the same sample with the scan rate of 1°C/min. Results from the DSC measurements were analyzed with the NanoAnalyze Software V 1.1 (TA Instruments, USA).

The samples contained 0.75 μg of *Fps*SSB, *Ppr*SSB and *Pto*SSB, 1 μg of *Dps*SSB, *Par*SSB and *Pcr*SSB, 1.5 μg of *Eco*SSB and 3 μg of *Pin*SSB were incubated at temperatures ranging from 60°C to 100°C for 0, 1, 2.5, 5, 10, 15, 30, 45, 60 min, after which, 0.05 pmol 5′-end fluorescein-labelled oligonucleotide (dT)_35_ was added. The samples were then loaded onto 2% agarose gels without ethidium bromide and separated by electrophoresis in a TAE buffer as described for EMSA tests. The incubation periods for each temperature, where 50% of (dT)_35_ was bound, were noted.

### Protein sequence analysis

The amino acid sequences of studied SSB proteins were analyzed using standard protein–protein BLAST and RPS-BLAST. Multiple sequence alignment was generated in ClustalX, using a PAM 500 scoring matrix. The results were prepared using the GeneDoc editor program (http://www.psc.edu/biomed/genedoc).

## Abbreviations

dsDNA: Double-stranded DNA; OB fold: Oligonucleotide/oligosaccharide binding fold; RPA: Replication protein A; SSB: Single-stranded DNA-binding; ssDNA: Single-stranded DNA.

## Competing interests

The authors declare that they have no conflict of interests.

## Authors’ contributions

MO conceived the study and carried out the molecular genetic studies. MN participated in the design of the study, carried out the molecular genetic studies and drafted the manuscript. JK participated in the design of study and drafted the manuscript. All the authors have read and approved the final manuscript.
